# A Roadmap for Automating Lineage Tracing to Aid Automatically Explaining Machine Learning Predictions for Clinical Decision Support

**DOI:** 10.2196/27778

**Published:** 2021-05-27

**Authors:** Gang Luo

**Affiliations:** 1 Department of Biomedical Informatics and Medical Education University of Washington Seattle, WA United States

**Keywords:** clinical decision support, database management systems, forecasting, machine learning, electronic medical records

## Abstract

Using machine learning predictive models for clinical decision support has great potential in improving patient outcomes and reducing health care costs. However, most machine learning models are black boxes that do not explain their predictions, thereby forming a barrier to clinical adoption. To overcome this barrier, an automated method was recently developed to provide rule-style explanations of any machine learning model’s predictions on tabular data and to suggest customized interventions. Each explanation delineates the association between a feature value pattern and an outcome value. Although the association and intervention information is useful, the user of the automated explaining function often requires more detailed information to better understand the patient’s situation and to aid in decision making. More specifically, consider a feature value in the explanation that is computed by an aggregation function on the raw data, such as the number of emergency department visits related to asthma that the patient had in the prior 12 months. The user often wants to rapidly drill through to see certain parts of the related raw data that produce the feature value. This task is frequently difficult and time-consuming because the few pieces of related raw data are submerged by many pieces of raw data of the patient that are unrelated to the feature value. To address this issue, this paper outlines an automated lineage tracing approach, which adds automated drill-through capability to the automated explaining function, and provides a roadmap for future research.

## Introduction

Machine learning has won almost all data science competitions [1] and is a hot topic these days. It is about computer algorithms that automatically learn from data, such as extreme gradient boosting, support vector machine, and random forest [2]. Using machine learning predictive models for clinical decision support has great potential in improving patient outcomes and reducing health care costs [3-10]. However, most machine learning models are black boxes that do not explain their predictions. This creates a barrier to clinical adoption. To overcome this barrier, we recently developed an automated method to offer rule-style explanations of any machine learning model’s predictions on tabular data and to suggest customized interventions without reducing the model’s performance measures [11-14]. Each rule-style explanation delineates the association between a feature value pattern and an outcome value. A feature is also called an independent variable. For the prediction of future emergency department (ED) visits or inpatient stays for asthma for a patient with asthma, one example of the explanation is as follows:

- The patient had 2 ED visits related to asthma in the prior 12 months
  AND the patient’s average respiratory rate recorded in the prior 12 months is >25 and ≤28 breaths per minute
  →the patient will likely have at least 1 ED visit or inpatient stay for asthma in the next 12 months [13,14].

An ED visit is related to asthma if the ED visit has an asthma diagnosis code. For the item in the explanation showing that the patient had 2 ED visits related to asthma in the prior 12 months, 1 intervention suggested by the automatic explanation method [12-14] is to apply control procedures that decrease the likelihood that the patient will need emergency care.

The association and intervention information provided by the automatic explanation method for machine learning predictions is useful. However, the user of the automated explaining function often requires more detailed information to better understand the patient’s situation and to aid in decision making. More specifically, consider a feature value on the left-hand side of a rule-style explanation that is computed by an aggregation function on the raw data. The user often wants to rapidly drill through to see certain parts of the related raw data producing the feature value. In the context of a relational database, these parts refer to the most relevant attributes of the most essential source tuples producing the feature value. Which attributes are most relevant and which source tuples are most essential depend on both the concrete feature type and the clinical decision support application’s need and are illustrated by several examples throughout this paper. The patterns embedded in these parts could provide additional information on the patient that was lost during the aggregation process to compute the feature value. This drill-through task is frequently difficult and time-consuming because the few pieces of related raw data are submerged by many pieces of raw data of the patient that are unrelated to the feature value. For example, as Table 1 shows, the list of encounters of a patient with asthma displayed on the standard interface of an electronic medical record system includes much information that is irrelevant to the feature value “2 of the number of ED visits related to asthma that the patient had in the prior 12 months.”

**Table 1:** An example list of encounters of a patient with asthma displayed on the standard interface of an electronic medical record system.^a^

Visit date	Primary diagnosis^b^	Visit type	Department	Provider	Facility
Dec 20, 2020	Cough (R05)	Outpatient	HMC^c^ family medicine clinic	John Smith	HMC
Dec 18, 2020	Dysphagia, unspecified (R13.10)	Outpatient	HMC family medicine clinic	David Wong	HMC
…	…	…	…	…	…
Oct 15, 2020	Cystitis, unspecified without hematuria (N30.90)	Inpatient	UWMC^d^ 8SE	Leslie Hurdle	UWMC
*Oct 12, 2020* ^e^	*Viral infection, unspecified (B34.9)*	*Emergency*	*HMC HEDUCC* ^f^	*Patricia Sward*	*HMC*
Oct 09, 2020	Dizziness and giddiness (R42)	Outpatient	HMC family medicine clinic	Eve Johnson	HMC
…	…	…	…	…	…
Feb 11, 2020	Posttraumatic stress disorder, unspecified (F43.10)	Outpatient	HMC psychotherapy clinic	Amy Jiang	HMC
*Feb 08, 2020*	*Syncope and collapse (R55)*	*Emergency*	*HMC HEDUCC*	*Peter Shavlik*	*HMC*
Feb 03, 2020	Headache, unspecified (R51.9)	Outpatient	HMC family medicine clinic	Jude Lake	HMC
…	…	…	…	…	…

^a^This example list is made based on a similar list seen in real electronic medical record data at the University of Washington Medicine.

^b^This column does not show up on the standard interface. This column is included because it will be discussed in this paper.

^c^HMC: Harborview Medical Center.

^d^UWMC: University of Washington Medical Center.

^e^For the feature value “2 of the number of emergency department visits related to asthma that the patient had in the prior 12 months,” the related rows in the list producing the feature value are marked in italics.

^f^HEDUCC: Harborview Emergency Department Urgent Care Center.

For instance, in the rule-style explanation shown above, the first item on the left-hand side is the feature value “2 of the number of ED visits related to asthma that the patient had in the prior 12 months.” Asthma may or may not be the primary diagnosis of either of these 2 visits. For this feature value, the user of the automated explaining function wants to see the relevant parts of these 2 visits (visit date, primary diagnosis, department handling the visit, admitting provider, facility where the visit occurred) in the reverse chronological order (see Table 2), like the way encounters are displayed on the standard interface of an electronic medical record system. The patterns embedded in these parts give additional information on the patient not shown by the feature value, such as the time between these 2 visits, how long ago these 2 visits occurred, the primary diagnoses in these 2 visits, and whether these 2 visits occurred at the same facility. However, finding these parts is nontrivial. As seen in real electronic medical record data at the University of Washington Medicine, Intermountain Healthcare, and Kaiser Permanente Southern California, the patient could have had over 100 encounters in the prior 12 months. Only a few of these encounters are ED visits, and even fewer of them are ED visits related to asthma. To find the ED visits of the patient in the prior 12 months, the user would need some manual effort even if aided by the search function for the electronic medical record system. To figure out which of these visits are related to asthma, a task with which the search function often cannot provide much help, the user would need much more manual effort.

**Table 2:** An example of the parts of the related raw data that should be displayed for a feature value.^a^

Visit date	Primary diagnosis	Department	Provider	Facility
Oct 12, 2020	Viral infection, unspecified (B34.9)	HMC^b^ HEDUCC^c^	Patricia Sward	HMC
Feb 08, 2020	Syncope and collapse (R55)	HMC HEDUCC	Peter Shavlik	HMC

^a^For the example list shown in Table 1 and the feature value “2 of the number of emergency department visits related to asthma that the patient had in the prior 12 months,” the parts that the user of the automated explaining function wants to see are in the related raw data producing the feature value.

^b^HMC: Harborview Medical Center.

^c^HEDUCC: Harborview Emergency Department Urgent Care Center.

In practice, numerous possible features computed by various aggregation functions on all kinds of longitudinal attributes in the electronic medical records could be used for predictive modeling and automatic explanation. Examples of such features include whether the most recent asthma diagnosis of the patient is a primary diagnosis, the patient’s average respiratory rate recorded in the prior 12 months, the total number of distinct asthma medications ordered for the patient in the prior 12 months, the total number of units of asthma relievers that were ordered for the patient in the prior 12 months and were neither systemic corticosteroids nor short-acting beta-2 agonists, the number of distinct asthma medication prescribers of the patient in the prior 12 months, and the number of no-shows by the patient in the prior 12 months [13,14]. Most of the possible features are unanticipated by the developers of the search function for the electronic medical record system beforehand. The search function supports only a few fixed types of search. For only a small portion of possible features, the search function can aid drilling through the raw data that produce a given feature value.

This creates a problem for the widespread adoption of the automatic explanation method for machine learning predictions. Frequently, this method gives multiple rule-style explanations for a patient predicted to be at high risk of incurring a poor outcome [11,12]. The user of the automated explaining function is typically a busy clinician having no time to do laborious manual drill-through regularly. However, to better understand the patient’s situation and to make better clinical decisions, the user often wants to drill through multiple feature values of the patient appearing in the explanations. If done manually, this is a challenging task. A patient often has extensive records with numerous variables and hundreds of pages of content accumulated over a long period of time [15]. Further, the relevant raw data producing the feature values are frequently scattered in several places in the electronic medical record system.

This study makes 2 contributions toward solving this problem:

1. We articulate this problem for the first time in the literature. This is done in the “Introduction” section.
2. To address this problem, an automated lineage tracing approach is outlined to add automated drill-through capability to the automated explaining function. This is done in the “Outline of the proposed automated lineage tracing approach” section. Further, a roadmap for future research is provided in the “Directions for future research” section.

The automated drill-through capability is intended to be offered to help the user of the automated explaining function save time, better understand the patient’s situation, and make better clinical decisions. The discussion in this paper focuses on structured electronic medical record data, a specific method commonly used to build clinical machine learning predictive models, and the automatic explanation method for machine learning predictions [11,12]. Nevertheless, the automated lineage tracing approach is not limited to them. Instead, when automatically explaining machine learning predictions and after appropriate extension, the principle of this approach can be applied to facilitate drilling through any feature value computed by an aggregation function on longitudinal structured data, regardless of whether the data came from electronic medical records, whether the feature is specified by a human expert or semiautomatically extracted from longitudinal data using the method outlined in the prior paper [16], which method is used to build the machine learning predictive model, or which automatic explanation method is used.

## Running Example

To illustrate this approach, a running example is used throughout this paper: automatically explaining the predictions of future ED visits or inpatient stays for individual patients with asthma. Our prior papers [12-14,17-19] detail this use case and the features used to make predictions in it.

### Base Tables

Below are the schemas of 5 tables in a relational database used in the running example:

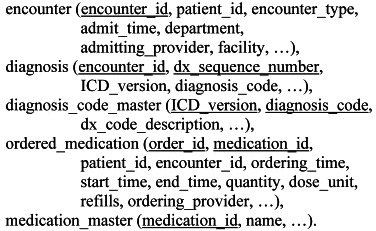



The underlined fields mark the key to each table. The *encounter* table includes 1 row per encounter listing its information. The *diagnosis* table includes 1 row per diagnosis code of an encounter. Primary diagnoses are signified by *dx_sequence_number*=1. The *diagnosis_code_master* table includes 1 row per unique diagnosis code giving its description. The *ordered_medication* table includes 1 row per medication appearing in a medication order. The *medication_master* table includes 1 row per unique medication listing its information.

### Intermediate Result Tables

Besides the above 5 base tables, 4 intermediate result tables computed on the new data are also used in the running example: *enc_features_1*, *enc_features_2*, *enc_features_3*, and *med_features_1*. The trained machine learning predictive model is applied to the new data to make predictions on individual patients.

The intermediate result table *enc_features_1* contains 3 temporal features on encounters: the number of ED visits, the number of inpatient stays, and the number of outpatient visits that the patient had in the prior 12 months. Let *today_date* denote today’s date. *enc_features_1* is computed from the *encounter* base table using the following structured query language (SQL) query.

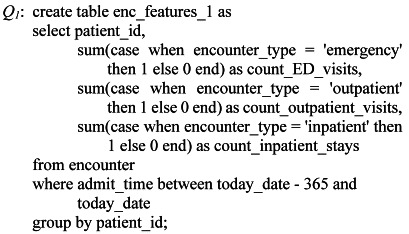



The intermediate result table *enc_features_2* contains 1 temporal feature on encounters: the number of outpatient visits with a primary diagnosis of asthma that the patient had in the prior 12 months. Recall that the International Classification of Diseases, Tenth Revision diagnosis codes of asthma are J45.x. *enc_features_2* is computed by joining the *encounter* and *diagnosis* base tables using the following SQL query.

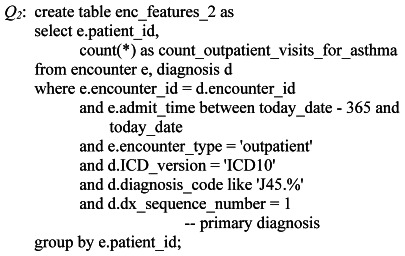



The intermediate result table *enc_features_3* contains 2 temporal features on encounters: the number of ED visits related to asthma and the number of inpatient stays related to asthma that the patient had in the prior 12 months. *enc_features_3* is computed by joining the *encounter* and *diagnosis* base tables using the following SQL query.

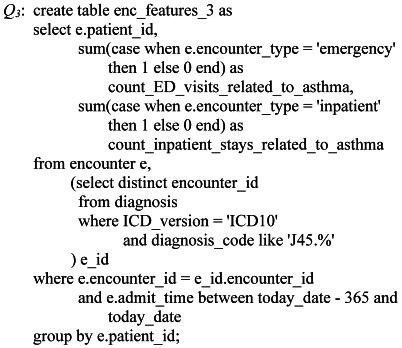



The intermediate result table *med_features_1* contains 2 temporal features on medications: the total number of medications and the total number of distinct medications ordered for the patient in the prior 12 months. *med_features_1* is computed from the *ordered_medication* base table using the following SQL query.

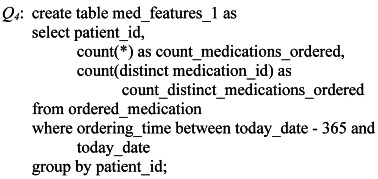



### Relational Algebra Operators

This paper uses the following relational algebra operators with the bag semantics unless otherwise specified: join 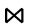, left semijoin 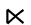, selection σ, projection π, duplicate elimination δ, and grouping γ [20]. Commercial database management systems implement relations using the bag semantics.

## Review of a Typical Method to Build a Clinical Machine Learning Predictive Model and Our Automated Method to Explain the Model’s Predictions

In this section, a typical method to build a machine learning predictive model on structured electronic medical record data as well as the automated method to explain the model’s predictions [11-14] are reviewed. In the next section, the automated lineage tracing approach based on these 2 methods is outlined.

A health care system usually has an enterprise data warehouse. It stores in a relational database a copy of the structured electronic medical record data of the health care system, often after some transformations such as pivoting [21,22] and denormalization to facilitate data analysis. For predictive modeling with automated explanation, the overall workflow is to execute database SQL queries to extract features from the electronic medical record data, to build a machine learning predictive model on the training data, to apply the model on new data to make predictions on individual patients, and then to use the automated method to explain the predictions. In the following sections, each of these steps is described sequentially.

### Extracting Features From the Electronic Medical Record Data and Building the Clinical Machine Learning Predictive Model

**Figure 1:**
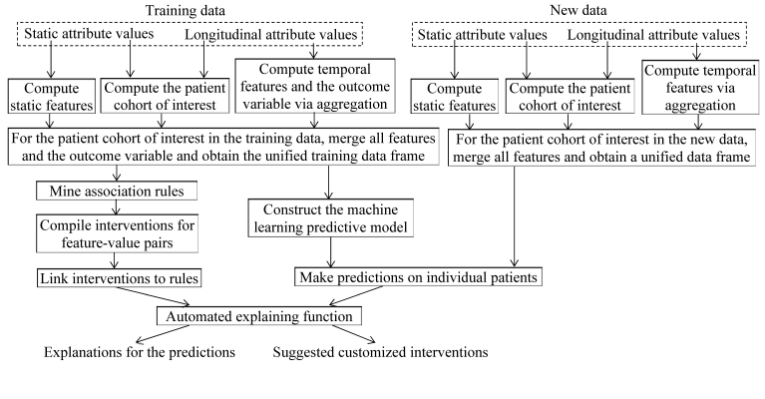
The flow chart for building a clinical machine learning predictive model on the training data, making predictions on the new data, and using our automated method to explain the model’s predictions.

The structured electronic medical record data contain both static attributes (eg, gender) and longitudinal attributes (eg, encounters, diagnoses). Most attributes are longitudinal. As Figure 1 shows, the following operations are performed on the training data:

1. The static features are computed from the static attribute values. The results are stored in 1 or more intermediate result tables. Typically, each of these intermediate result tables is computed by running a select-project-join SQL query on 1 or more base tables.
2. By aggregating longitudinal attribute values and sometimes also using some static attribute values, the patient cohort of interest in the training data is computed. The result is stored in 1 intermediate result table. This is typically done by running a complex SQL query on several base tables. An example patient cohort is the set of all patients with asthma who visited any of the facilities of the health care system during a specific time period.
3. By aggregating longitudinal attribute values, temporal features and the outcome variable are computed and stored in 1 or more intermediate result tables. Typically, each of these intermediate result tables is computed by running a select-project-join-aggregate SQL query on 1 or more base tables. For example, 1 intermediate result table is similar to *enc_features_1* and contains multiple temporal features on encounters computed from the *encounter* base table. A second intermediate result table is similar to *enc_features_2* and contains multiple temporal features on encounters computed by joining the *encounter* and *diagnosis* base tables. A third intermediate result table contains multiple temporal features on medications computed by joining the *ordered_medication* and *medication_master* base tables, such as the total number of distinct asthma medications and the total number of units of asthma medications ordered for the patient in the prior 12 months. The logical query plan for a select-project-join-aggregate query includes 1 or more select-project-join-aggregate segments [23]. Each segment has a grouping or duplicate elimination operator at its end following a bunch of join, selection, and projection operators.

**Figure 2:**
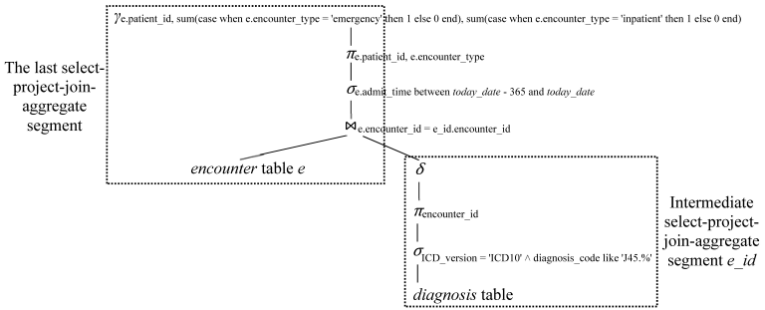
A logical query plan for the select-project-join-aggregate query *Q_3_* given in the “Intermediate result tables” section.

Figure 2 shows the logical query plan for a select-project-join-aggregate query. By joining the intermediate result tables containing the patient cohort of interest, the static and temporal features, and the outcome variable in the training data, a table containing the unified training data frame is obtained. For the patient cohort of interest, this table includes 1 column for the outcome variable and a separate column for each feature. Then a machine learning predictive model is trained on this table.

### Applying the Machine Learning Predictive Model to New Data to Make Predictions on Individual Patients

**Figure 3:**
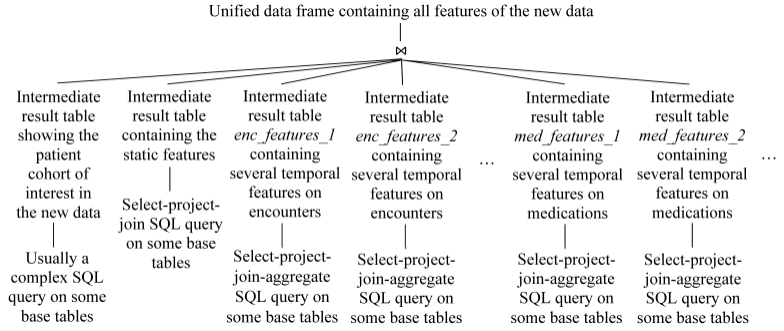
The high-level logical query plan for computing the unified data frame that contains all the features of the new data. SQL: structured query language.

As Figure 3 shows, similar to the procedure mentioned above, the patient cohort of interest and the static and temporal features in the new data are computed. The results are stored in several intermediate result tables. By joining these tables, a table containing the unified data frame for the new data is obtained. For the patient cohort of interest, this table includes a separate column for each feature. We then apply the machine learning predictive model to this table to make predictions on individual patients.

### Automatically Explaining the Machine Learning Model’s Predictions

At the same time of building the clinical machine learning predictive model, the training data are used to create the knowledge base of the automated explaining function. We do automated discretization [24,25] to convert continuous features to categorical features. Then class-based association rules [24,26] are mined from the unified training data frame. Each rule delineates the association between a feature value pattern and a poor outcome value *c* and is of the form

*i_1_* AND *i_2_* AND … AND *i_t_*→*c*.

This rule shows that a patient satisfying *i_1_*, *i_2_*, …, and *i_t_* tends to have an outcome value *c*. The values of *t* and *c* can change across rules. Each item *i_k_* (1≤*k*≤*t*) is a (feature, value) pair showing that a feature has a specific value or a value within a specific range. One example item of the former is that the patient had 2 ED visits related to asthma in the prior 12 months. One example item of the latter is that the patient’s average respiratory rate recorded in the prior 12 months is >25 and ≤28 breaths per minute. An example rule containing both items is given in the Introduction.

For each (feature, value) pair item used to create association rules, 0 or more interventions are precompiled. The interventions precompiled for any item on a rule’s left-hand side are automatically linked to the rule.

At prediction time, to avoid reducing the machine learning predictive model’s performance measures, the model’s predictions are used with no change. The mined association rules are used to explain these predictions rather than to make predictions. More specifically, for each patient whom the model predicts to have a poor outcome value, we find and display the rules that have this value on their right-hand sides and whose left-hand sides are fulfilled by the patient. Each rule offers 1 explanation for the prediction. The interventions linked to the rule are displayed next to it as the suggested candidate interventions.

Our automatic explanation method for machine learning predictions has been successfully applied to multiple clinical predictive modeling problems [11,12,27,28]. It has several advantages. Among all the automatic explanation methods for machine learning predictions in the literature [29,30], our method is the only one that can automatically suggest customized interventions. The rule-style explanations given by our method are easier to comprehend than the non–rule-style explanations given by many other methods. Unlike many other automatic explanation methods that either lower the machine learning predictive model’s performance measures or work for only a specific machine learning algorithm, our automatic explanation method works for any machine learning algorithm on tabular data without lowering the model’s performance measures. Unlike several other methods that use rules computed at prediction time to offer explanations [31,32], our method uses rules mined before prediction time to offer explanations. This is essential for our method to automatically suggest customized interventions at prediction time.

## Review of the Existing Automated Lineage Tracing Techniques

In this section, the existing automated lineage tracing techniques are reviewed. An overview of such techniques developed in various fields is provided. Then, a specific set of automated lineage tracing techniques most closely related to this work is reviewed.

### Overview of the Existing Automated Lineage Tracing Techniques

The lineage or provenance of a given data item *i* refers to the source data items producing *i* and how *i* was derived [33]. The former is called where-lineage. The latter is called how-lineage. Each type of lineage can be at either the schema level or the instance level. An example of where-lineage at the schema level is the set of base tables producing a specific materialized view. An example of where-lineage at the instance level is the set of tuples in the base tables producing a given temporal feature value in a materialized view. Lineage information can be computed in either an eager way or a lazy way. In the former case, lineage information is computed and stored at the same time of producing the output data. In the latter case, lineage information is computed when needed. This paper focuses on where-lineage that is at the instance level and computed in a lazy way.

Ikeda et al surveyed existing lineage tracing techniques in databases [33,34], e-science [35], and scientific data processing [36]. Among all of the lineage tracing techniques in the literature, the techniques Cui et al [23,37] developed are the most closely related to this work. These techniques are used to trace the lineage of a tuple in a materialized view [38] defined by a select-project-join-aggregate query in a relational database. Cui et al [39,40] described lineage tracing techniques for warehouse data computed via a directed acyclic graph of transformations, some of which could involve complex procedural code. Zhang et al [41] described lineage tracing techniques for data computed by arbitrary functions. In general, the more flexibility is allowed on the transformations or functions, the less efficiently lineage can be traced [39].

In big data systems, Ikeda et al [42,43] described lineage tracing techniques for data computed via a directed acyclic graph of map and reduce functions [44]. Amsterdamer et al [45] described lineage tracing techniques for data computed by using Pig Latin [46].

In scientific data processing, lineage tracing is often done on curated databases, which contain scientific data copied from other databases [36,47].

Schelter et al [48] described a method to trace the schema-level lineage of the data sets, features, models, and predictions produced in machine learning experiments.

### Review of Cui et al’s Automated Lineage Tracing Techniques for Relational Databases

To automatically trace the lineage of a tuple *t* in a materialized view [38] defined by a select-project-join-aggregate query, Cui et al [23,37] proceeded as follows. First, the materialized view’s definition query is transformed into a canonical form of the logical query plan. As Figure 2 shows, the canonical form includes 1 or more select-project-join-aggregate segments. Each segment has 0 or 1 join operator, 0 or 1 selection operator, 0 or 1 projection operator, and a grouping or duplicate elimination operator in this particular order. Second, a separate intermediate materialized view is created for each intermediate select-project-join-aggregate segment of the canonical form. The root node of such a segment is not the root node of the canonical form. Third, we recursively trace through the hierarchy of intermediate materialized views in a top-down way. At each level of the hierarchy, the lineage tracing query for a 1-level select-project-join-aggregate materialized view is used to compute the current traced tuples’ lineage with respect to each base table and each materialized view at the next lower level. For a 1-level select-project-join-aggregate materialized view *MV* = *γ*(*π_A_*(*σ_C_*(*R_1_*
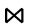
*R_2_*
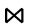
…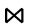 *R_n_*))), the lineage of a tuple set *T*⊆*MV* with respect to the base table or the materialized view *R_i_* (1≤*i*≤*n*) is *π_Ri_*(*σ_C_*(*R_1_* 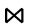 *R_2_* 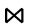
…
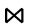
*R_n_*)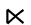 *T*). Here, the projection operator *π* on *R_i_* has the set semantics, making each selected tuple in *R_i_* appear only once. Further, all attributes of *R_i_* appear in the projection operator and subsequently in the lineage traced on *R_i_*. The final traced lineage of tuple *t* includes the lineage traced on every base table appearing in the canonical form.

We use an example to illustrate Cui et al’s [23,37] automated lineage tracing techniques. If “create table enc_features_3” is replaced by “create materialized view enc_features_3_view” in query *Q_3_* given in the “Intermediate result tables” section, a query *Q_3_v_* defining a materialized view *enc_features_3_view* is obtained. To trace the lineage of a tuple *t* in *enc_features_3_view* whose *patient_id* is *asthma_patient_id*, one proceeds as follows.

First, the canonical form of the logical query plan for query *Q_3_v_* is obtained. The canonical form is the same as the logical query plan for query *Q_3_* shown in Figure 2.

Second, an intermediate materialized view *asthma_encounter_id* is created for the intermediate select-project-join-aggregate segment *e_id* shown in Figure 2. This is done using the following SQL query.

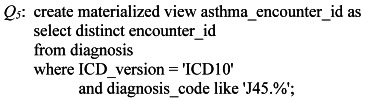



**Figure 4:**
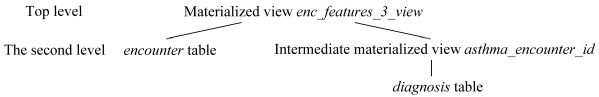
The hierarchy of intermediate materialized views matching the canonical form of the logical query plan for the definition query of the materialized view *enc_features_3_view*.

Figure 4 shows the resulting hierarchy of intermediate materialized views, with the materialized view *enc_features_3_view* at the top and the *encounter* and *diagnosis* base tables at the bottom.

Third, at the top level of the hierarchy of intermediate materialized views, the lineage of tuple *t* with respect to the *encounter* base table is computed using the following SQL query.

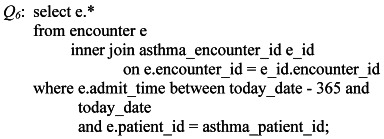



The following SQL query is used to compute the lineage of tuple *t* with respect to the intermediate materialized view *asthma_encounter_id* and to store the results in a temporary table *temp*.

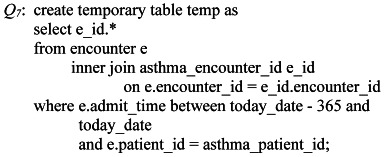



Fourth, at the second level of the hierarchy of intermediate materialized views, the lineage of the tuples in the temporary table *temp* with respect to the *diagnosis* base table is computed using the following SQL query.

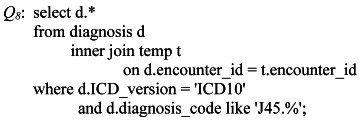



The final traced lineage of tuple *t* includes both the results of query *Q_6_* and the results of query *Q_8_*.

## Outline of the Proposed Automated Lineage Tracing Approach

In this section, an automated lineage tracing approach is outlined to add automated drill-through capability to the automated explaining function. Our presentation includes 4 subsections. In the first subsection, an overview of the lineage tracing component of the automated explaining function is provided. In the second subsection, the unique requirements on automated lineage tracing are shown for automatically explaining machine learning predictions for clinical decision support. In the third subsection, the proposed automated lineage tracing techniques fulfilling these requirements is outlined. In the fourth subsection, some considerations are presented for future computer coding implementation of the proposed lineage tracing approach.

### Overview of the Lineage Tracing Component

At association rule mining time, all (feature, value) pair items used to create association rules are known. Which items involve temporal features computed by aggregation functions on the raw data is also known. For each item that is related to a temporal feature of a patient and on the left-hand side of a rule, a hyperlink is added to the item in the rule. In addition, a parameterized stored procedure is written for the item in the database to retrieve lineage information. The stored procedure typically has 2 parameters: the *patient_id* of the patient being examined and the endpoint of the temporal aggregation period, such as today. When the stored procedure is run for the first time, an execution plan is generated. All subsequent runs will use the same execution plan to avoid runtime query optimization overhead.

At automatic explanation time, the user of the automated explaining function is allowed to do lineage tracing for any item that is on the left-hand side of a rule-style explanation and related to a temporal feature value. When the user clicks the item’s hyperlink, the stored procedure prewritten for the item is invoked to retrieve some prespecified parts of the related raw data producing the feature value. Except for the cases with 2 specific aggregation functions described later in the paper, the retrieved data instances are always displayed on a page in the reverse chronological order like that in the electronic medical records.

### Unique Requirements for Automated Lineage Tracing

Typically, the user of the automated explaining function is a clinician. To fit the user’s busy schedule and to aid timely decision making, the user wants the lineage tracing process for a temporal feature value to be finished quickly, preferably within 1 second. This goal is partially fulfilled by the existing lineage tracing techniques [23,37], whereas the realized lineage tracing speed can be further improved. In addition, the retrieved lineage information should be easy to scan and include the most essential content needed to facilitate decision making. This enables the user to quickly gain useful insights from the information, ideally within 1 or a few seconds. As summarized in Table 3, that goal translates to 5 unique requirements on automated lineage tracing that are unmet by the existing lineage tracing techniques.

**Table 3:** The 5 unique requirements of automated lineage tracing for automatically explaining machine learning predictions for clinical decision support.

Requirement	Reason for posing the requirement
Retrieving only a small set of attributes	To prevent the user from being overwhelmed by many nonessential or irrelevant attributes
Adding some essential attributes that do not directly produce the feature value	To make the retrieved lineage information include the most essential content
Sorting the retrieved lineage information in an appropriate order	To make the retrieved lineage information easy to scan
Computing the lineage information based on the semantic meaning of the feature	To avoid including irrelevant or nonessential source tuples in the retrieved lineage information
Performing no lineage tracing for any health care system feature value computed by an aggregation function	To avoid including irrelevant data in the retrieved lineage information

#### Requirement 1: Retrieving Only a Small Set of Attributes

When tracing the lineage of a temporal feature value, one should retrieve from the base tables only a small set of attributes specific to the temporal feature rather than the many attributes involved in deriving all of the features used for automated explanation. This requirement is posed to prevent the user of the automated explaining function from being overwhelmed by many nonessential or irrelevant attributes.

To aid automatic explanation, we want to allow tracing the lineage of a temporal feature value in the form of a small set of attributes specific to the temporal feature (see Table 2 for an example). This cannot be well done using Cui et al’s lineage tracing techniques [23,37]. These techniques were developed to trace the lineage of a tuple including all of its attribute values in a select-project-join-aggregate materialized view in a relational database. If the retrieved lineage information ever touches a tuple in a base table, all attribute values of the tuple are included in this information. For automatic explanation, both factors would cause the retrieved lineage information to have an excessive volume, overwhelming the user of the automated explaining function.

To see this, the process of making predictions with automatic explanations is reviewed. Usually, many features are used to make predictions and to automatically explain them. All of the items on the left-hand side of a rule-style explanation come from the same tuple in the unified data frame, which contains all features of the new data. As Figure 3 shows, this unified data frame is obtained by joining many intermediate result tables. Each of them falls into 1 of the 3 categories: (1) a table containing the patient cohort of interest in the new data, (2) a table containing 1 or more static features, and (3) a table containing 1 or more temporal features. Each hyperlinked item on the left-hand side of a rule-style explanation comes from exactly 1 intermediate result table in the third category.

When the user of the automated explaining function clicks the hyperlink for an item on the left-hand side of a rule-style explanation, one could use Cui et al’s techniques [23,37] to trace the lineage of the tuple in the unified data frame, from which the item comes. For each intermediate result table mentioned above and each base table used to create it, the retrieved lineage information contains some tuples from the base table including all of their attribute values. Most of the retrieved lineage information is unnecessary for automatic explanation for 3 reasons.

##### Reason 1

The retrieved lineage information often includes thousands of tuples from several dozen base tables. Most of these base tables are used to compute the other feature values in the tuple in the unified data frame that are unrelated to the item, and include no information that can help the user of the automated explaining function gain useful insights related to the item. In fact, to obtain the lineage information of the item essential for automatic explanation, we need to only trace through the intermediate result table related to the item solely for the item and to examine the base tables used to create this table. The features in this table that are unrelated to the item can be ignored. There is also no need to trace through the intermediate result tables containing the features unrelated to the item. Moreover, at automatic explanation time, we know the *patient_id* of the patient linked to the item. The user usually does not need to know why this patient is in the patient cohort of interest in the new data. Thus, there is no need to trace through the intermediate result table showing the patient cohort.

##### Reason 2

A base table often has many attributes, only a few of which are essential for the user of the automated explaining function to gain useful insights related to the item. For instance, the *encounter* table often has >100 attributes. The lineage information shown in Table 2 covers only 4 of them: *admit_time* transformed to the date format, *department*, *admitting_provider*, and *facility*.

##### Reason 3

Certain items are each computed using several base tables and intermediate query results. For the user of the automated explaining function to gain useful insights related to the item, only the attributes and tuples of some of these base tables are essential. Alternatively, none or only some of these intermediate query results need to be traced through.

For example, in query *Q_2_* given in the “Intermediate result tables” section, both the *encounter* and *diagnosis* base tables are used to compute the feature “the number of outpatient visits with a primary diagnosis of asthma that the patient had in the prior 12 months.” For a value of this feature, we need to use the information in the *diagnosis* table to find the related tuples in the *encounter* table. Nevertheless, the user would expect each encounter shown in the retrieved lineage information to be an outpatient visit with a primary diagnosis of asthma. Thus, there is no need to include any attribute or tuple from the *diagnosis* table in the retrieved lineage information, for example, to give the primary diagnosis of each encounter included in that information.

As a second example, in query *Q_3_* given in the “Intermediate result tables” section, both the *encounter* base table and the intermediate query result *e_id* are used to compute the feature “the number of ED visits related to asthma that the patient had in the prior 12 months.” For a value of this feature, the user of the automated explaining function would expect each encounter shown in the retrieved lineage information to be an ED visit related to asthma, like that shown in Table 2. Thus, there is no need to trace through *e_id* and to obtain the corresponding tuples in the *diagnosis* table showing that each encounter included in the retrieved lineage information has an asthma diagnosis code.

#### Requirement 2: Adding Some Essential Attributes That Do Not Directly Produce the Feature Value

For certain temporal features, when acquiring the lineage of a feature value, one should not use only the related raw data that directly produce the feature value. Instead, one needs to add to them some related attributes in the base tables, which are specific to the temporal feature and do not directly produce the feature value. We pose this requirement to make the retrieved lineage information include the most essential content needed to facilitate decision making. For example, as query *Q_1_* given in the “Intermediate result tables” section shows, the feature “the number of ED visits that the patient had in the prior 12 months” is computed solely from the *encounter* base table. For a value of this feature, we want the retrieved lineage information to be similar to that shown in Table 2 and include a primary diagnosis column. This column is computed using the *diagnosis* and *diagnosis_code_master* base tables unused in *Q_1_* and is formed by concatenating the *diagnosis_code* and *dx_code_description* columns of the *diagnosis_code_master* base table. The cases for many other temporal features on encounters are similar.

#### Requirement 3: Sorting the Retrieved Lineage Information in an Appropriate Order

When presenting the lineage information, the related raw data retrieved for a temporal feature value should be sorted in an order specific to the temporal feature. This requirement is posed to make the retrieved lineage information easy to scan. Usually, we want the data instances in the retrieved lineage information to be displayed in the reverse chronological order like that in the electronic medical records. However, there are 2 exceptions. First, when the temporal feature is the maximum value of an attribute of a given patient, we want the related raw data retrieved for a feature value to be displayed in the descending order of the attribute value. For example, for the feature “the highest systolic blood pressure of the patient in the prior 12 months,” we want the lineage information retrieved for a feature value to contain the systolic blood pressure of the patient in the prior 12 months sorted in the descending order. Second, when the temporal feature is the minimum value of an attribute of a given patient, we want the related raw data retrieved for a feature value to be displayed in the ascending order of the attribute value. In either of the 2 cases, a resort button could be added to the retrieved lineage information on display. If the user of the automated explaining function clicks this button, the data instances in the retrieved lineage information are rearranged in the reverse chronological order for display.

#### Requirement 4: Computing the Lineage Information Based on the Semantic Meaning of the Feature

The lineage information of a temporal feature value should be computed based on the semantic meaning of the feature rather than solely on the literal writing of the SQL query used to compute the feature. We pose this requirement to avoid including irrelevant or nonessential source tuples in the retrieved lineage information. For a select-project-join-aggregate materialized view containing 1 or more temporal features, Cui et al [23,37] compute the lineage of a tuple in it based solely on the literal SQL query used to define it. In certain cases, this literal approach is suboptimal for automatic explanation. Instead, we should consider the semantic meanings of the temporal features during lineage tracing. In the following, 2 such cases are described. Each case is presented as a subrequirement.

##### Subrequirement 4.1

When the temporal feature is the sum of a variable computed by a case statement in SQL including multiple conditions and some of them return 0, only the lineage information related to the other conditions should be retrieved. In SQL, such a temporal feature is written in the form of

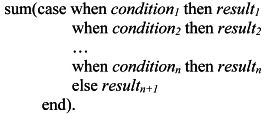



As an example of this subrequirement, for the feature “the number of ED visits that the patient had in the prior 12 months,” the lineage information retrieved for a value of the feature should be the ED visits that the patient had in the prior 12 months, regardless of whether the feature is computed using SQL query *Q_9_* or *Q_10_* below.

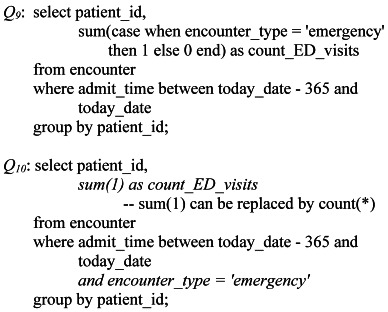



The differences between *Q_9_* and *Q_10_* are highlighted in italics in *Q_10_*. If the feature is computed using *Q_9_*, Cui et al’s techniques [23,37] would retrieve all the encounters of the patient in the prior 12 months as the lineage information. This could easily overwhelm the user of the automated explaining function, as usually most of these encounters are not ED visits.

##### Subrequirement 4.2

When the temporal feature is the total number of distinct items, the retrieved lineage information should include only 1 representative data instance for each distinct item. For example, query *Q_4_* given in the “Intermediate result tables” section computes the feature “the total number of distinct medications ordered for the patient in the prior 12 months.” For a value of this feature, Cui et al’s techniques [23,37] would retrieve all medications ordered for the patient in the prior 12 months as the lineage information. This information is often overwhelming and not succinct enough for the user of the automated explaining function to quickly find the distinct medications ordered for the patient in the prior 12 months, as the same medication could be ordered for the patient multiple times in a year. To avoid this problem, one could retrieve only the most recent order of each distinct medication ordered for the patient in the prior 12 months as the lineage information. For the user, these distinct medications typically provide enough insight into the patient’s status related to the feature value.

#### Requirement 5: Performing No Lineage Tracing for Any Health Care System Feature Value Computed by an Aggregation Function

We do not trace the lineage of any health care system feature value computed by an aggregation function. We pose this requirement to avoid including irrelevant data in the retrieved lineage information. Like temporal features of a patient, certain health care system features [17-19] such as the number of patients with asthma of the primary care provider of a patient are computed by aggregation functions. These health care system features are each computed using multiple patients’ information rather than solely the information of the patient being examined. Since other patients’ detailed information does not help the user of the automated explaining function understand this patient’s situation, we do not trace the lineage of any value of this feature, even if it appears on the left-hand side of a rule-style explanation.

### Outline of the Proposed Techniques to Form the Lineage Tracing Query That Computes the Lineage Information

To perform automated lineage tracing for explaining machine learning predictions for clinical decision support, Cui et al’s lineage tracing techniques [23,37] are modified to fulfill the requirements mentioned above. Even without giving any detail on the computer coding implementation and the performance evaluation results, Cui et al [37] already used 49 pages to describe the details of their automated lineage tracing algorithm. The case described in this paper is more complex than Cui et al’s case [37]. In the case described in this paper, which attributes are most relevant and which source tuples are most essential for inclusion in the retrieved lineage information depend on both the concrete feature type and the clinical decision support application’s need. In comparison, no such dependency exists in Cui et al’s case [37]. Thus, it is expected that, once fully worked out, the proposed automated lineage tracing algorithm would be more sophisticated than Cui et al’s algorithm [37]. In this viewpoint paper, the goal is not to enumerate all possible feature types and to provide a detailed design or any computer coding implementation of the proposed automated lineage tracing approach. Rather, the goal is to describe the design approach for the proposed automated lineage tracing module and to provide a roadmap for future research. We achieve this goal by outlining the main steps of forming the lineage tracing query, giving 4 example temporal features, and illustrating at a high level how to form the lineage tracing query for each of these 4 features.

#### Overview of the Lineage Tracing Query Formation Process

Usually, each intermediate result table shown in Figure 3 has a *patient_id* column. It is used as the join column in the join operation to produce the unified data frame containing all features of the new data. As explained in “Reason 1” of the “Requirement 1” section, to obtain the lineage information of a temporal feature value, we need to only trace through the intermediate result table containing this value solely for this value. This intermediate result table is usually computed from some base tables by using a select-project-join-aggregate SQL query *S_0_*. To form the lineage tracing query for a temporal feature value of a patient in the intermediate result table, one proceeds in 4 steps. First, the other temporal features, if any, are removed from *S_0_* to obtain a simplified query *S_1_*. Second, if applicable, *S_1_* is transformed to query *S_2_* to fulfill subrequirement 4.1. Third, Cui et al’s techniques [23,37] are modified to address Reasons 2 and 3 given in the “Requirement 1” section. The modified techniques are used to form a preliminary lineage tracing query *S_3_* based on *S_2_* and the patient’s *patient_id*. Fourth, to obtain the final lineage tracing query, *S_3_* is transformed to fulfill Requirements 2 and 3 and subrequirement 4.2.

In the following, 4 examples are used to illustrate at a high level how to form the lineage tracing query. In each example, the user of the automated explaining function is examining a patient with asthma whose identifier is *asthma_patient_id* and wants to drill through a temporal feature value of this patient. We outline the main steps of forming the lineage tracing query for the feature value without giving the detailed algorithm.

#### Example 1: The Number of ED Visits That the Patient Had in the Prior 12 Months

As defined by query *Q_1_* in the “Intermediate result tables” section, the intermediate result table *enc_features_1* contains 3 temporal features. One of them is the number of ED visits that the patient had in the prior 12 months. To form the lineage tracing query for a value of this feature, one proceeds as follows.

First, the other 2 features are removed from query *Q_1_* to obtain query *Q_9_* given in the “Subrequirement 4.1” section.

Second, to fulfill subrequirement 4.1 on handling the sum of a variable computed by a case statement, query *Q_9_* is transformed to query *Q_10_* given in the “Subrequirement 4.1” section.

Third, Cui et al’s lineage tracing techniques [23,37] are used to form a draft lineage tracing query *Q_11_* based on *Q_10_* and *asthma_patient_id*.

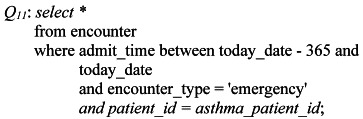



The differences between *Q_10_* and *Q_11_* are highlighted in italics in *Q_11_*. To address Reason 2 given in the “Requirement 1” section and retrieve from the *encounter* table only its attributes essential for automatic explanation, *Q_11_* is transformed to the following preliminary lineage tracing query.

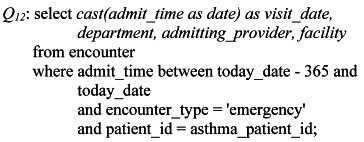



The differences between *Q_11_* and *Q_12_* are highlighted in italics in *Q_12_*.

Fourth, to fulfill Requirement 2, a primary diagnosis column needs to be added to the raw data that are retrieved by query *Q_12_* and that directly produce the feature value being examined. To fulfill Requirement 3, the retrieved raw data need to be sorted in the reverse chronological order. To meet both demands, *Q_12_* is transformed to the following final lineage tracing query.

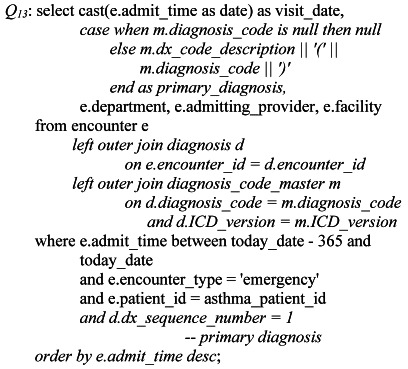



The differences between *Q_12_* and *Q_13_* are highlighted in italics in *Q_13_*. || is the string concatenation operator in SQL.

#### Example 2: The Number of Outpatient Visits With a Primary Diagnosis of Asthma That the Patient Had in the Prior 12 Months

As defined by query *Q_2_* in the “Intermediate result tables” section, the intermediate result table *enc_features_2* contains the temporal feature “the number of outpatient visits with a primary diagnosis of asthma that the patient had in the prior 12 months.” To form the lineage tracing query for a value of this feature, one proceeds as follows.

First, to address Reason 2 given in the “Requirement 1” section, only the attributes essential for automatic explanation should be included from the *encounter* table. To address Reason 3 given in the “Requirement 1” section, no attribute or tuple from the *diagnosis* table should be included in the retrieved lineage information. A preliminary lineage tracing query *Q_14_* is formed based on query *Q_2_* and *asthma_patient_id* by using a modified version of Cui et al’s lineage tracing techniques [23,37] that meets both demands.

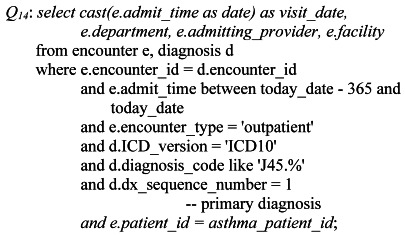



The differences between *Q_2_* and *Q_14_* are highlighted in italics in *Q_14_*.

Second, to fulfill Requirement 3 of sorting the related raw data retrieved for the feature value in the reverse chronological order, query *Q_14_* is transformed to the following final lineage tracing query.

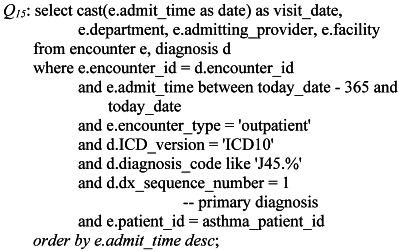



The differences between *Q_14_* and *Q_15_* are highlighted in italics in *Q_15_*.

#### Example 3: The Number of ED Visits Related to Asthma That the Patient Had in the Prior 12 Months

As defined by query *Q_3_* in the “Intermediate result tables” section, the intermediate result table *enc_features_3* contains 2 temporal features. One of them is the number of ED visits related to asthma that the patient had in the prior 12 months. To form the lineage tracing query for a value of this feature, one proceeds as follows.

First, the other feature is removed from query *Q_3_* to obtain the following simplified query.

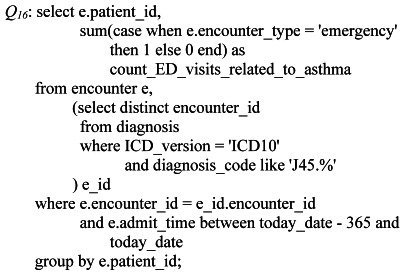



Second, to fulfill subrequirement 4.1 on handling the sum of a variable computed by a case statement, query *Q_16_* is transformed to the following query.

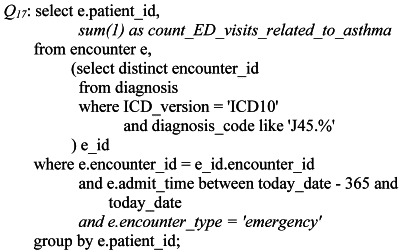



The differences between *Q_16_* and *Q_17_* are highlighted in italics in *Q_17_*.

Third, to address Reason 2 given in the “Requirement 1” section, only the attributes essential for automatic explanation should be included from the *encounter* table. To address Reason 3 given in the “Requirement 1” section, the intermediate query result *e_id* should not be traced through to include any corresponding tuple in the *diagnosis* table in the retrieved lineage information. A preliminary lineage tracing query *Q_18_* is formed based on query *Q_17_* and *asthma_patient_id* by using a modified version of Cui et al’s lineage tracing techniques [23,37] that meets both demands.

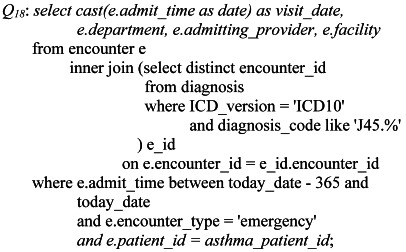



The differences between *Q_17_* and *Q_18_* are highlighted in italics in *Q_18_*.

Cui et al’s lineage tracing techniques [23,37,49] are applied to query *Q_3_* to create a materialized view *asthma_encounter_id*, which is defined by query *Q_5_* in the “Review of Cui et al’s automated lineage tracing techniques for relational databases” section. The *asthma_encounter_id* is used to rewrite the preliminary lineage tracing query *Q_18_* as follows.

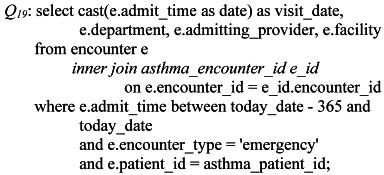



The differences between *Q_18_* and *Q_19_* are highlighted in italics in *Q_19_*.

Fourth, to fulfill Requirement 2, a primary diagnosis column needs to be added to the raw data that are retrieved by query *Q_19_* and that directly produce the feature value being examined. To fulfill Requirement 3, the retrieved raw data need to be sorted in the reverse chronological order. To meet both demands, *Q_19_* is transformed to the following final lineage tracing query.

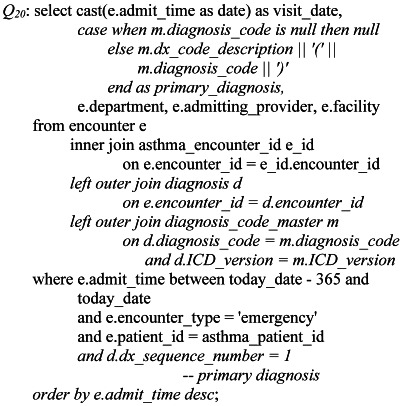



The differences between *Q_19_* and *Q_20_* are highlighted in italics in *Q_20_*.

#### Example 4: The Total Number of Distinct Medications Ordered for the Patient in the Prior 12 Months

As defined by query *Q_4_* in the “Intermediate result tables” section, the intermediate result table *med_features_1* contains 2 temporal features. One of them is the total number of distinct medications ordered for the patient in the prior 12 months. To form the lineage tracing query for a value of this feature, one proceeds as follows.

First, the other feature is removed from query *Q_4_* to obtain the following simplified query.

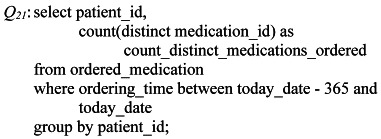



Second, to address Reason 2 given in the “Requirement 1” section, only the attributes essential for automatic explanation should be included from the *ordered_medication* table. A preliminary lineage tracing query *Q_22_* is formed based on query *Q_21_* and *asthma_patient_id* by using a modified version of Cui et al’s lineage tracing techniques [23,37] that meets this demand.

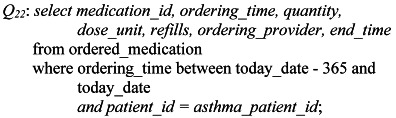



The differences between *Q_21_* and *Q_22_* are highlighted in italics in *Q_22_*.

Third, to fulfill subrequirement 4.2, one could retrieve only the most recent order of each distinct medication ordered for the patient in the prior 12 months as the lineage information. This is done by transforming query *Q_22_* to the following query.

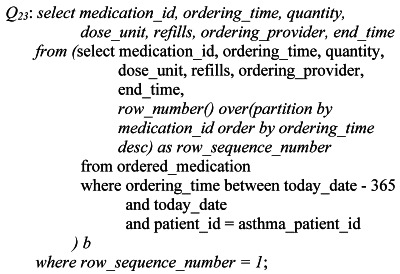



The differences between *Q_22_* and *Q_23_* are highlighted in italics in *Q_23_*.

Fourth, to fulfill requirement 2, a medication name column is added to the raw data that are retrieved by query *Q_23_* and directly produce the feature value being examined. To fulfill Requirement 3, the retrieved raw data are sorted in the reverse chronological order. *Q_23_* is transformed to the following final lineage tracing query to meet both demands.

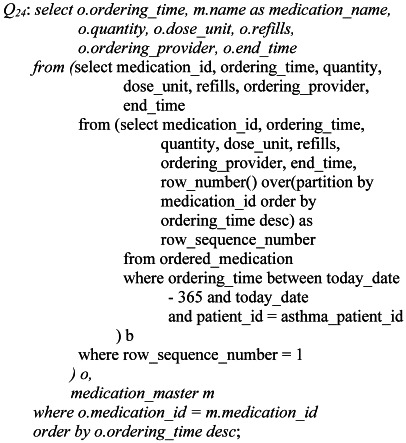



The differences between *Q_23_* and *Q_24_* are highlighted in italics in *Q_24_*.

### Considerations for Future Computer Coding Implementation of the Proposed Automated Lineage Tracing Approach

#### Maximizing the Automation Degree of the Lineage Tracing Query Formation Process

For a select-project-join-aggregate materialized view, Cui et al [23,37] used a fully automated approach to analyze its definition query to derive a lineage tracing query for a tuple in it. In the case of automatically explaining machine learning predictions, all temporal features used for making predictions and automatic explanation are known at machine learning model building time. In general, for each temporal feature, we can form a lineage tracing query either manually or semiautomatically, but often not fully automatically, beforehand. Nevertheless, once the query is formed and put into the knowledge base of the automated explaining function, we can use the query to automatically retrieve the lineage information of a value of the feature at prediction time.

As mentioned before, automatic explanation poses several unique requirements on automated lineage tracing. Two of them make it difficult to fully automate the lineage tracing query formation process. First, Requirement 1 says that the lineage information retrieved for a temporal feature value should include only a small set of relevant attributes specific to the temporal feature. Almost infinite attributes and temporal features could possibly be used for clinical machine learning. Thus, it is infeasible to precompile the set of relevant attributes for every possible temporal feature. Second, Requirement 2 says that when acquiring the lineage of a value for certain temporal features, we need to include some attributes that are specific to the temporal feature and do not directly produce the feature value. For a reason similar to the above, it is infeasible to precompile the set of such attributes for every possible such temporal feature.

Although the lineage tracing query formation process cannot be fully automated in the most general case, 2 methods can still be used to maximize the process’ automation degree and to reduce the workload of the developers of the automated explaining function. First, for a temporal feature, an approach similar to that of Cui et al [23,37] can be used to automatically form a draft lineage tracing query. The developers of the automated explaining function revise this query as needed to obtain the final lineage tracing query. Second, the same temporal feature is often used for multiple predictive modeling tasks. One can create a library of lineage tracing queries for temporal features to facilitate query reuse across various predictive modeling tasks. This library is formed for a data set in the Observational Medical Outcomes Partnership common data model format [50] using its linked standardized terminologies [51]. This format standardizes administrative and clinical variables from ≥10 large US health care systems [52,53]. For any data set that is put into this format, we can use this library to obtain lineage tracing queries.

#### Improving the Lineage Tracing Speed

As mentioned before, the user of the automated explaining function wants the lineage tracing process for a temporal feature value to be finished quickly, preferably within 1 second. To expedite tracing the lineage of a tuple in a materialized view defined by a select-project-join-aggregate query *S*, Cui et al [23,37,49] advocated creating a materialized view for each intermediate select-project-join-aggregate segment of the canonical form of the logical query plan for *S*. While this boosts the lineage tracing speed, the resulting speed is still not fast enough to reach a subsecond response time [23,39]. To further improve the lineage tracing speed, we can build indices [39,42] on the selection and join attributes of both the base tables and the materialized views created for the intermediate select-project-join-aggregate segments. For instance, in Example 3, we can build 1 index on the *encounter_id* column of the materialized view *asthma_encounter_id* and another index on the *patient_id* column of the *encounter* base table. We can create indices either manually or by using an automated index design tool provided by a commercial relational database system [54-56]. Typically, each intermediate result table containing 1 or more temporal features is computed on 1 or a few base tables using no more than a small number of join operations. The lineage tracing query for a temporal feature value falls into a similar case. Thus, with appropriate indices, we would expect the lineage tracing query to finish execution quickly. For base tables of moderate sizes and simple materialized views, Cui and Widom [39] showed that lineage tracing can be done within 1 second when indices exist on the keys of the base tables. For large base tables and temporal features computed through more complex procedures, we would expect that more indices are needed to reach a subsecond response time.

The above discussion focuses on the case that the electronic medical record data are stored in a relational database and features are extracted using SQL queries. When the electronic medical record data are stored in a big data system and features are extracted using map and reduce functions [44] or Pig Latin [46], we can modify the corresponding existing lineage tracing techniques [42,43,45] in a similar way to enable lineage tracing to aid automatically explaining machine learning predictions for clinical decision support.

## Discussion

### Directions for Future Research

The above discussion describes the high-level design approach for the proposed automated lineage tracing module. To complete the detailed design of the proposed automated lineage tracing approach, implement the module in computer code, and test the module’s performance, much research is needed along the following directions:

1. We need to compile a list of attributes and temporal feature types most commonly used in building clinical machine learning predictive models. For these attributes and temporal feature types, we need to complete the detailed design and the computer coding implementation of the proposed automated lineage tracing approach.
2. We need to come up with an automated approach to design indices needed for improving the lineage tracing speed. The database research community has developed several automated index design approaches [54-56]. We can modify these approaches to fit the database querying workload posed by automated lineage tracing.
3. We plan to assess the execution speed of the proposed automated lineage tracing approach after implementing it in computer code.
4. As shown by prior work on automated lineage tracing shown in the “Overview of the existing automated lineage tracing techniques” section, the database research community takes it for granted that automated lineage tracing could help users better understand the data and save time in doing data analysis. To the best of our knowledge, no formal study to date has been published on measuring the impact of automated lineage tracing on users’ data analysis and decision-making process. After implementing the proposed automated lineage tracing module, we plan to choose several clinical predictive modeling tasks and assess for each task, the impact of offering the module on the data analysis and decision-making process of the users of the automated explaining function. In particular, we plan to evaluate whether the addition of the module benefits the user and improves outcomes, for example, by saving the user’s time, making it easier for the user to understand the predictions given by the machine learning predictive model and helping the user better understand the patient’s situation and make better clinical decisions.

### Limitations of the Proposed Approach

The proposed automated lineage tracing approach has several limitations:

1. To build clinical machine learning predictive models, we usually use temporal features that are computed by SQL queries of low or moderate complexities. It is possible that some temporal features used to build certain predictive models are computed by rather complex SQL queries. We may not be able to finish the lineage tracing process for a value of such a temporal feature quickly, regardless of how many indices are built to expedite this process. For example, this could happen if the SQL query uses complex procedural code, which has no property that can be used to simplify the lineage tracing process [39]. Having a long lineage tracing time could make the user of the automated explaining function become impatient. Nevertheless, it is still faster and more convenient to do lineage tracing using the automated approach than to let the user do manual drill-through.
2. The proposed automated lineage tracing approach works for any feature values computed by the standard aggregation functions in SQL on longitudinal structured data. For certain deep learning predictive models built on longitudinal structured data, the previously proposed method [16] could be used to semiautomatically extract comprehensible and predictive temporal features from the models and the longitudinal structured data, and then apply the automated approach to trace the lineage of the values of these features. For any other deep learning predictive model that is built directly on longitudinal structured data and that uses incomprehensible features hidden in the neurons of the deep neural network, the proposed automated approach can no longer be used to trace the lineage of the values of these features.
3. Almost infinite attributes and temporal features could possibly be used for clinical machine learning. Further, some attributes are not covered by the Observational Medical Outcomes Partnership common data model. For the reasons given in the “Maximizing the automation degree of the lineage tracing query formation process” section, we could maximize the automation degree of the lineage tracing query formation process for only certain types of temporal features formed on certain attributes. For any other temporal feature, the developers of the automated explaining function could still need a nontrivial amount of time to create the corresponding lineage tracing query.

### Conclusions

Automatically explaining machine learning predictions is critical to overcome the model interpretability barrier to using machine learning predictive models in clinical practice. Our previously developed automatic explanation method for machine learning predictions can be used to address this barrier, but a gap remains to fulfill the need of rapidly drilling through a feature value in an explanation that is computed by an aggregation function on the raw data. This paper articulates this gap, outlines an automated lineage tracing approach to close the gap, and provides a roadmap for future research. The automated drill-through capability is intended to be offered to help the user of the automated explaining function save time, better understand the patient’s situation, and make better clinical decisions. It would take several people multiple years to work out the detailed design and the computer coding implementation of the proposed automated lineage tracing approach. We hope this paper will make some researchers become interested in and join the research endeavor on this topic. Only after the detailed design and the computer coding implementation of the proposed automated lineage tracing approach are fully worked out, one could deploy the automated lineage tracing module in clinical practice and measure the module’s impact on clinicians’ decision-making process. The principle of the automated lineage tracing approach generalizes to nonmedical data and other automated methods to explain machine learning predictions.

## Abbreviations

ED: emergency department
SQL: structured query language
